# Supplementary material for: Learning curve and surgical outcome of robotic assisted colorectal surgery with ERAS program
Source: Sci Rep. 2022 Nov 29;12:20566. doi: 10.1038/s41598-022-24665-w (PMC9709162; doi:10.1038/s41598-022-24665-w)
Supplement: Supplementary file 1 — Supplementary Information 1. [file 41598_2022_24665_MOESM1_ESM.zip › LSC Raw data-Ñ[▒K/148/P_ñw╝╨░O▒Kñσ.pdf]

[redacted]

Pathologic diagnosis:
Colon, terminal ileum, and appendix, right hemicolectomy --- a. Adenocarcinoma, moderately differentiated, of cecum. Tumor involved orifice of appendix, ileocecal valve and distal ileum, invaded to mesocolic soft tissue and subserosal area, and metastasized to mesocolic lymph nodes (4/18). The bilateral cut ends and mesenteric lymph node (0/1) are free of tumor. b. Non-caseating granulomatous inflammation in the submucosal area of ileum.

Ancillary study for diagnosis:
IHC stains for PMS2 and MSH6 are done on section H. Both above markers are preserved.

Prognostic and predictive factor:
1. Tumor size: 7x4x2 cm.
2. Depth of invasion: pericolorectal tissue.
3. Histologic grade: G2: Moderately differentiated.
4. Angiolymphatic invasion: Not identified.
5. Perineural invasion: Not identified.
6. Tumor deposits (discontinuous extramural extension): Present, specify number of deposits: 1.
7. Macroscopic tumor perforation: Not identified.
8. Surgical Margins: a) Proximal Margin: Uninvolved by invasive carcinoma, distance of tumor from margin: 14 cm. b) Distal Margin: Uninvolved by invasive carcinoma, distance of tumor from margin: 24 cm. c) Radial or Mesenteric Margin: Cannot be assessed.
9. Lymph node status: Involved (4/19).
10. Extranodal involvement: Present.
11. Tumor regression grading S/P CCRT: No known presurgical therapy.
12. Type of polyp in which invasive carcinoma arose: Not identified.
13. Additional pathologic findings: Not identified.
14. Pathological TNM Stage: pT3N2a (According to the eighth edition, American Joint Committee on Cancer Staging Guidelines for Tumors).
15. TNM descriptors: Not applicable.

Gross description:
The specimen consists of a segment of colon, 26 cm in length, and 5.5 cm in proximal circumference, 8 cm in distal cut end circumference, and a segment of terminal ileum, 15 cm in length, and 4 cm in cut end circumference. An unremarkable appendix, 4x1x0.7 cm, is noted. A gray tan firm annular elevated and centrally ulcerative tumor, 7x4x2 cm, is noted over cecum and ileocecal valve, 14 cm to the proximal cut end and 24 cm to the distal cut end. The ileocecal valve is severely deformed and barely recongnized. On cross section, the tumor invaded to the mesocolon with serosal retraction noted. Two pieces of omentum, 15x11x2 cm and 9x4x1 cm, are noted and they are adherent to tumor. The orifice of appendix and distal ileum are involved by tumor. Bilateral cut ends free of tumor. Representative part for section: A) proximal cut end B) distal cut end C-D) tumor and mesocolon E) tumor and omentum F) tumor and small intestine G) tumor and orifice of appendix H-J) tumor and colon K-P) mesocolic lymph nodes Q) mesenteric lymph nodes.

Note:
1. The interpretation of MMR panel and recommended studies are as below: (1) No loss of nuclear expression of MMR proteins: low probability of microsatellite instability-high (MSI-H). (2) Loss of nuclear expression of MLH1 and PMS2: testing for methylation of the MLH1 promoter and/or mutation of BRAF is indicated (the presence of a BRAF V600E mutation and/or MLH1 methylation suggests that the tumor is sporadic and germline evaluation is probably not indicated; absence of both MLH1 methylation and of BRAF V600E mutation suggests the possibility of Lynch syndrome, and sequencing and/or large deletion/duplication testing of germline MLH1 may be indicated). (3) Loss of nuclear expression of MSH2 and MSH6: high probability of Lynch syndrome (sequencing and/or large deletion/duplication testing of germline MSH2 may be indicated, and, if negative, sequencing and/or large deletion/duplication testing of germline MSH6 may be indicated). (4) Loss of nuclear expression of MSH6 only: high

indicated). (4) Loss of nuclear expression of MSH6 only: high
probability of Lynch syndrome (sequencing and/or large
deletion/duplication testing of germline MSH6 may be indicated). (5)
Loss of nuclear expression of PMS2 only: high probability of Lynch
syndrome (sequencing and/or large deletion/duplication testing of
germline PMS2 may be indicated). (6) Please note: There are
exceptions to the above IHC interpretations. These results should not
be considered in isolation, and clinical correlation with genetic
counseling is recommended to assess the need for germline testing.
2. This case has been peer reviewed by two doctors.
#T-59420_2 #M-81403_2 2 1105 S0000F
#T-C4560_2 #M-81406_2 2 1105 S00000
#T-59200_2 #M-09183_2 0 1105 000000

已電子簽章

敬告：本單僅供醫師參考使用，不具任何申請效力！！
請病患妥為保管，若不慎遺失需自負個人資料外洩責任！
